# Isolation and characterization of urine microvesicles from prostate cancer patients: different approaches, different visions

**DOI:** 10.1186/s12894-021-00902-8

**Published:** 2021-09-27

**Authors:** María García-Flores, Christian M. Sánchez-López, Marta Ramírez-Calvo, Antonio Fernández-Serra, Antonio Marcilla, José Antonio López-Guerrero

**Affiliations:** 1 Laboratory of Molecular Biology, Fundación Instituto Valenciano de Oncología, 46009 Valencia, Spain; 2 IVO-CIPF Joint Research Unit of Cancer, Príncipe Felipe Research Center (CIPF), 46012 Valencia, Spain; 3 Àrea de Parasitologia, Departament de Farmàcia i Tecnologia Farmacèutica i Parasitologia, Universitat de València, 46000 Burjassot, Valencia, Spain; 4 Joint Research Unit on Endocrinology, Nutrition and Clinical Dietetics, Health Research Institute La Fe, Universitat de Valencia, 46100 Valencia, Spain; 5 Department of Pathology, School of Medicine, Catholic University of Valencia “San Vicente Mártir”, 46001 Valencia, Spain

**Keywords:** Extracellular microvesicles, Exosomes, Ultracentrifugation, Size exclusion chromatography, miRNA

## Abstract

**Background:**

Because of their specific and biologically relevant cargo, urine extracellular vesicles (EVs) constitute a valuable source of potential non-invasive biomarkers that could support the clinical decision-making to improve the management of prostate cancer (PCa) patients. Different EV isolation methods differ in terms of complexity and yield, conditioning, as consequence, the analytical result.

**Methods:**

The aim of this study was to compare three different isolation methods for urine EVs: ultracentrifugation (UC), size exclusion chromatography (SEC), and a commercial kit (Exolute® Urine Kit). Urine samples were collected from 6 PCa patients and 4 healthy donors. After filtered through 0.22 µm filters, urine was divided in 3 equal volumes to perform EVs isolation with each of the three approaches. Isolated EVs were characterized by spectrophotometric protein quantification, nanoparticle tracking analysis, transmission electron microscopy, AlphaScreen Technology, and whole miRNA Transcriptome.

**Results:**

Our results showed that UC and SEC provided better results in terms of EVs yield and purity than Exolute®, non-significant differences were observed in terms of EV-size. Interestingly, luminescent AlphaScreen assay demonstrated a significant enrichment of CD9 and CD63 positive microvesicles in SEC and UC methods compared with Exolute®. This heterogeneity was also demonstrated in terms of miRNA content indicating that the best correlation was observed between UC and SEC.

**Conclusions:**

Our study highlights the importance of standardizing the urine EV isolation methods to guaranty the analytical reproducibility necessary for their implementation in a clinical setting.

**Supplementary Information:**

The online version contains supplementary material available at 10.1186/s12894-021-00902-8.

## Background

The diagnosis of prostate cancer (PCa) is currently made by histological confirmation from a prostate biopsy guided by altered serum prostate-specific antigen (PSA) values (≥ 4 ng/ml) and/or a suspicious digital rectal examination (DRE) [1–3]. However, this approach presents many limitations including low specificity of PSA and DRE and the molecular heterogeneity of PCa that at the end determines tumour behavior [4]. For this reason, there is an urgent need in developing more targeted and non-invasive diagnostic tools, based on the molecular characterization of body fluids, that provide information about the malignant potential of PCa and allowing the monitoring of the disease into the different clinical scenarios.

Urine, due to the anatomic proximity of the prostate gland to the urethra, constitutes a valuable source of PCa biomarkers particularly derived from exfoliated prostatic cells, excreted proteins, circulating nucleic acids or extracellular vesicles (EVs) [5]. EVs are small membrane vesicles that are classified according to their size, cellular origin and biogenesis into microvesicles, exosomes, and apoptotic bodies [6, 7]. They are released by most cell types in physiological and pathological conditions [7] and can be isolated from all body fluids (including urine, blood, saliva, milk, semen, cerebrospinal fluid, etc.) [8, 9]. EVs contains a variety of molecules including nucleic acids, proteins, lipids, and some other metabolites [10–12], and their composition is affected by different environmental factors and health status [13, 14]. Given their ability to horizontally transfer genetic material and signaling moieties between different cells in the organism, EVs have recently emerged as powerful mediators of cell–cell communication [7].

Currently, EVs-cargo represents a doubtless source of biomarkers that may represent the different PCa progression stages [15, 16] and constitute promising tools for the development of minimally invasive diagnostic approaches. Hence, because of their increasing potential for their use in clinical scenarios, it has become vitally important to improve the isolation methods for maximum purity, yield, and assay reproducibility [17]. The most common approaches for EVs isolation include size exclusion chromatography (SEC); classical ultracentrifugation (UC) [17]; sucrose density-gradient centrifugation; affinity chromatography using antibodies against EVs markers (such as CD9, CD81, CD63) [18]; or commercial kits [8, 19–21]. Despite their importance, EVs isolation and characterization are still considered major scientific challenges [22, 23], and identifying the best techniques for their isolation is crucial for further biomarker discoveries.

The aim of this study was to compare three different EVs isolation methods: UC, SEC, and a commercial kit (Exolute® Urine Kit) using urine from a series of PCa patients and healthy donors (HDs). The outperformance of the three methods was evaluated by using different analytic approaches, including NanoDrop protein quantification, nanoparticle tracking analysis (NTA), transmission electron microscopy (TEM), AlphaScreen Technology, and HTG EdgeSeq miRNA Whole Transcriptome Assay (miRNA WTA).

## Methods

### Sample collection and ethical considerations

Urine samples from six PCa patients and four HDs (men with no history of cancer or other prior chronical diseases), were retrieved from the archives of the Biobank of the Fundación Instituto Valenciano de Oncología (FIVO). Written informed consent for sample donation for research purposes was obtained from all patients prior to sample collection, and the study was approved by the Clinical Research Ethics Committee (CREC) and the Institutional Ethics Committee (Ref. PROMETEO 2016/103), at the meeting held on May 28, 2015. All methods used during the study were performed in accordance with the relevant guidelines and regulations.

### Urine processing

A median of 72 mL (range: 54–90 mL) of urine were collected in sterile urine containers (Ref. 409726 Deltalab, Barcelona, Spain). Protease Inhibitor Cocktail (P8340-5 mL, Sigma Aldrich, San Luis, MO, USA) was added to preserve exosomes (50 µL cocktail in 100 mL urine sample) [24]. Each sample was centrifuged at 1000× *g*, 10 min at 4 °C, and supernatant were frozen at − 80 °C until use. The pre-analytical variables of the samples with SPREC code [25–27] are shown in Table 1.

**Table 1:** Preanalytical variables included in the Standard PREanalytical Code (SPREC), applied to urine samples

Sample ID	Type of sample	SPREC code^a^
127	PCa patient	URN-PIX-D-D-N-N-J
129	PCa patient	URN-PIX-D-D-N-N-J
132	PCa patient	URN-PIX-A-D-N-N-J
144	PCa patient	URN-PIX-B-D-N-N-J
146	PCa patient	URN-PIX-B-D-N-N-J
148	PCa patient	URN-PIX-B-D-N-N-J
161	HDs	URN-PIX-A-D-N-N-J
163	HDs	URN-PIX-A-D-N-N-J
164	HDs	URN-PIX-A-D-N-N-J
167	HDs	URN-PIX-A-D-N-N-J

^a^Each biospecimen is assigned a seven-element-long code that corresponds to seven preanalytical variables. First code element: type of sample (URN: urine). Second code element: type of primary container (PIX: with Protease inhibitors). Third code element: precentrifugation (A: RT < 2 h, B: 3–7 °C < 2 h, D: 3–7 °C 2–4 h). Fourth code element: centrifugation (D: 3–7 °C 10 min < 3000 g with braking). Fifth code element: second centrifugation (N: No centrifugation). Sixth code element: postcentrifugation (N: Not applicable). Seventh code element: storage condition [J: PP (Poly propylene) tube ≥ 5 mL (− 85) to (− 60) °C. If the preanalytical option used is unknown or inconstant, the letter “X” is used. If the preanalytical option used is known but does not correspond to any of the standard options, the letter “Z” is used. (RT: room temperature 18–25 °C) [25]

Briefly, urine samples were thawed at 4 °C before use. Samples were centrifuged at 1000× *g*, 15 min at room temperature (RT) to remove cell debris, and the collected supernatants were then centrifuged at 3000× *g*, 15 min at 4 °C. The major urinary contaminant, mucoprotein (the Tamm-Horsfall protein), was removed by adding NaCl to a final concentration of 0.58 M and incubated for 2 h at RT, as previously described [28]. Samples were then centrifuged at 16,000× *g*, for 20 min at 4 °C, and the supernatant was collected and filtered through 0.22 µm membrane filters (Thermo Fisher Scientific, Waltham, MA, USA). Finally, the total volume of urine from each sample was divided into 3 equal parts, to perform the 3 different EVs isolation methods.

### Isolation methods

*EVs enrichment by ultracentrifugation (UC)* Urine supernatants were centrifuged at 100,000× *g*, 2 h at 4 °C (in a 50.2 Ti Titanium rotor, Beckman coulter, using a CP100NX ultracentrifuge, Hitachi). The 100,000× *g* pellet was resuspended in 150 µL of filtered Phosphate-Buffered Saline (PBS).

*Size exclusion chromatography (SEC)* EVs isolation was performed as described by Böïng et al. [29]. Briefly, up to 12 mL of Sepharose-CL2B (Sigma-Aldrich, San Luis, MO, USA) were stacked in a 15 mL syringe (Sigma-Aldrich, San Luis, MO, USA), and washed 3 times with PBS, previously filtered through 0.22 µm membrane filters, and used as elution buffer. Samples were concentrated using Amicon® Ultra-4 Centrifugal Filter Devices (EMD Millipore, Burlington, MA, USA) by centrifugation at 3200× *g*, 20 min at 4 °C. Then, up to 0.75 mL of concentrated urine was loaded into the column, and a total of 20 fractions of 0.5 mL were collected from each sample. Fractions 8 and 9 were pooled and selected for further EVs analysis.

*ExoLutE® Urine Kit* Urine supernatants were concentrated to a final volume of 7 mL using Amicon® Ultra-4 Centrifugal Filter Devices (EMD Millipore Burlington, Ma, USA), and then processed with ExoLutE® Urine Kit (Rosetta Exosome® Inc., Seoul, Republic of Korea) following the manufacturer’s instructions. A total of 130 µL of EVs-enriched samples were obtained.

### Characterization methods

*NanoDrop quantification* The protein concentration in each sample was measured at 280 nm absorbance using a NanoDrop 1000 spectrophotometer (Thermo Scientific, Waltham, MA, USA).

*Nanoparticle tracking analysis (NTA)* Size distribution of particles was determined by NTA in a NanoSight LM10 (Malvern Instrument Ltd, Malvern, UK), using a 405 nm laser and sCMOS camera. Data were analyzed with using the NTA software version 3.3 (Dev Build 3.3.104), with Min track Length, Max Jump Distance, and Blur set to auto, and detection threshold set to 5. Camera level was set to 15, and 5 readings of 30 s at 30 frames per second were taken with manual monitoring of temperature. Samples were diluted with filtered PBS to reach the concentration (20–120 particles/frame) recommended by the manufacturer***.***

*Transmission electron microscopy (TEM)* Sample preparation was performed as already described [30] with modifications. Briefly, 8 µL of EVs-containing samples were fixed in 2% paraformaldehyde (PFA) for 30 min, and deposited on Formvar-carbon coated EM grids for 15 min. Then, samples were washed with PBS 0, 1 M, and post-fixed with 1% Glutaraldehyde for 5 min, washed with distilled water, and then contrasted in a mixture of uranyl acetate (1%) and methyl cellulose (0.5%). Samples were analyzed with a Jeol JEM1010 TEM, operating at 80 kV. Four different samples were analyzed for each of the isolation methods, and 100 vesicles were counted in each sample. Images were recorded on a MegaView III digital camera, and EVs size was determined using the Olympus Image Analysis Software. The sample analysis was performed at the Microscopy facility of the Central Service for Experimental Research (SCSIE) from the University of Valencia.

*AlphaScreen™ Technology* Five µL of EVs resuspended in PBS were transferred to a 96-well white 1/2 area microplate (Perkin Elmer, Madrid, Spain). Samples were incubated overnight at 4 °C with 10 µL/well of anti-human CD9 antibody (SHI-EXO-M01-50; CosmoBio Co, Tokyo, Japan) conjugated to AlphaLisa acceptor beads (10 µg/mL; 6,772,001, Perkin Elmer, Madrid, Spain), and 10 µL/well of biotinylated human anti-CD63 antibody (3 nM, SHI-EXO-M02-50, CosmoBio Co, Tokyo, Japan) previously biotinylated. Then, 25 µL/well of AlphaScreenTM streptavidin-coated donor beads (40 µg/mL; 6,760,002, Perkin Elmer, Madrid, Spain) were added and incubated in the dark for another 30 min at RT. A signal appears (excitation spectra at 680 nm, emission spectra at 615 nm) if the distance between both beads is less than 200 nm (compatible with exosomes size and other small EVs) thanks to the reactivity of O2, and is detected using a Multifunctional microplate reader CLARIOstar® (BMG LABTECH, Ortenberg, Germany). Assays were carried out at Centro de Investigación Principe Felipe (CIPF), Valencia, Spain.

*HTG EdgeSeq miRNA Whole Transcriptome Assay (miRNA WTA)* Whole miRNA transcriptome expression analysis was performed with urine derived EVs of PCa patients and HDs using the HTG EdgeSeq System (HTG Molecular Diagnostics, Inc., Tucson, AZ, USA) with the HTG EdgeSeq miRNA Whole Transcriptome Assay (miRNA WTA). This assay quantified the expression of 2083 human RNA transcripts (https://www.htgmolecular.com/assays/mirna-wta).

The same amount of EVs (3,6 × 10^9^) from each patient were digested with proteinase K following the manufacturer’s instructions. miRNAs of interest were then selected by a protection nuclease assay (qNPA) in the HTG EdgeSeq processor using miRNA Whole Transcriptome Assay (miRNA WTA) panel (HTG Molecular Diagnostics, Inc., Tucson, AZ, USA). Library prepared with the miRNA selected was amplified by PCR using adapters for NextSeq 550 System sequencer (Illumina, San Diego, CA, USA). Samples were purified using Agencourt AMPure XP beads kit (Beckman Coulter, Brea, CA, USA), and then quantified by ABI 7500 Fast Real-Time PCR System (qPCR) using KAPA Library Quant Kit Universal qPCR Mix (KAPA Biosystems, Wilmington, MA, USA). For the qPCR, six standards were used in triplicate. Negative controls for PCR and qPCR were also used. After library quantification, all samples were normalized to a concentration of 20 pM. Library sequencing was performed using Next Generation Sequencing (NGS) in a NextSeq 550 System, High Output, 75 cycle v2.5 (Illumina, San Diego, CA, USA). Sequenced data were processed in HTG Parser Software using Bowtie2 for sequenced reads alignment.

### Statistical analysis

Kruskal–Wallis non-parametric test and U- Mann Whitney test for post-hoc pairwise comparisons were used to perform a statistical analysis of the different characterization methods. All statistical inference was performed with two-tailed tests with a significance level of 5%.

Additionally, in the processing of HTG EdgeSeq data, Principal Component Analysis was used for data visualization and DESeq2 method for the study of differentially expressed miRNAs. The IBM SPSS Statistics V22.0 package (SPSS, Chicago, IL), R v. 4.0.1 and GraphPad Prism 7 (GraphPad Software Inc.) were used for statistical analysis [31].

## Results

### Spectrophotometric quantification and NTA

To compare protein and particle yields between the three EVs-isolation procedures, a spectrophotometric quantification at 280 nm, as well as the determination of particle concentration and size distribution by NTA, was performed. Absorbance measurements showed similar protein concentrations in UC (7.57 ± 8.2 µg/mL) and SEC (7.468 ± 3.77 µg/mL), but a significant increase was noted in Exolute® samples (25.99 ± 19.25 µg/mL) (Additional file 1: Figure S1A). Additional file 1: Figure S1B shows the amount of protein divided into PCa patients and HDs, no significant differences were found between the two groups in any of the three isolation methods.

**Fig. 1:**
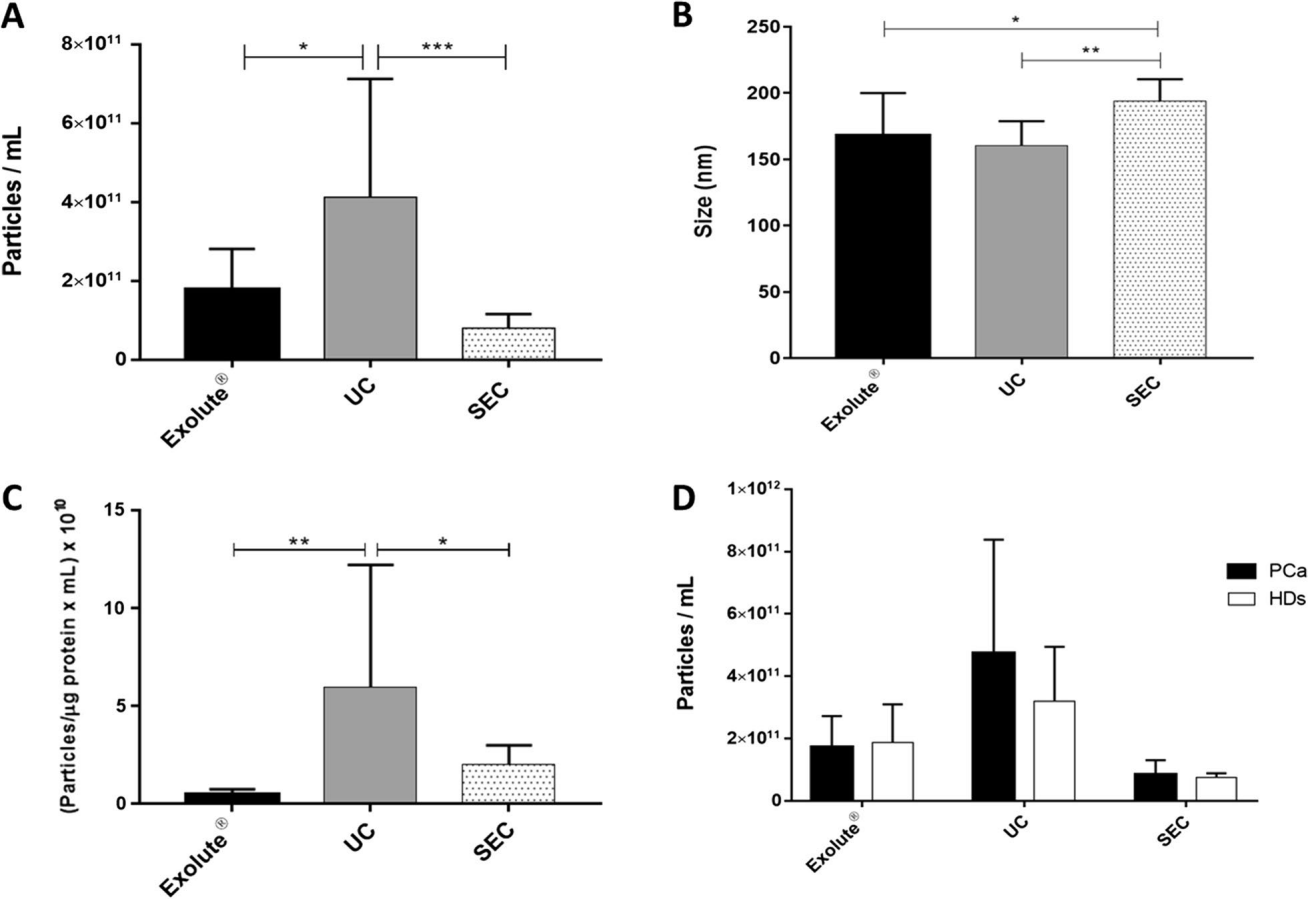
Characterization of EVs by NTA. **A** Concentration and **B** size of the EVs. **C** Purity of sample calculated by ratio between the number of particles per mL and µg of proteins/mL. **D** The concentration of EVs is similar between PCa and HDs with the three isolation methods employed, with the number of EVs being higher in UC, followed by Exolute® and SEC. (*p-value < 0.05; **p-value < 0.001; ***p-value < 0.0001)

NTA measurements revealed that UC provide the highest number of particles per mL (4.13 ± 3 × 10^11^ particles/mL) in comparison with Exolute® (1.78 ± 1.03 × 10^11^ particles/mL) *p*-value = 0.021, and SEC (8.1 ± 3.54 × 10^10^ particles/mL) *p*-value = 0.0011 (Fig. 1A). However, whereas Exolute® and UC particles had a similar size distribution (modes of 167.84 ± 32.19 and 160.28 ± 18.51 nm, respectively; *p*-value > 0.99), SEC particles showed larger size (194.1 ± 16.4 nm) (*p*-value = 0.0026) (Fig. 1B).

The ratio between the number of particles/mL and the µg of proteins/mL, as an estimation of the purity of the sample, was calculated the highest ratio being for UC samples (5.97 ± 6.24 × 10^10^), followed by SEC (2.01 ± 0.97 × 10^10^) and Exolute® (0.44 ± 0.28 × 10^10^), respectively (Fig. 1C).

Non-significant differences between PCa patients and HDs were observed. EVs isolated from UC and SEC showed a similar pattern, with higher particle and protein concentration in samples derived from PCa patients, which was inverted in Exolute®-EVs samples (Fig. 1D and Additional file 1: Figure S1B).

### Characterization of isolated EVs by transmission electron microscopy

**Fig. 2:**
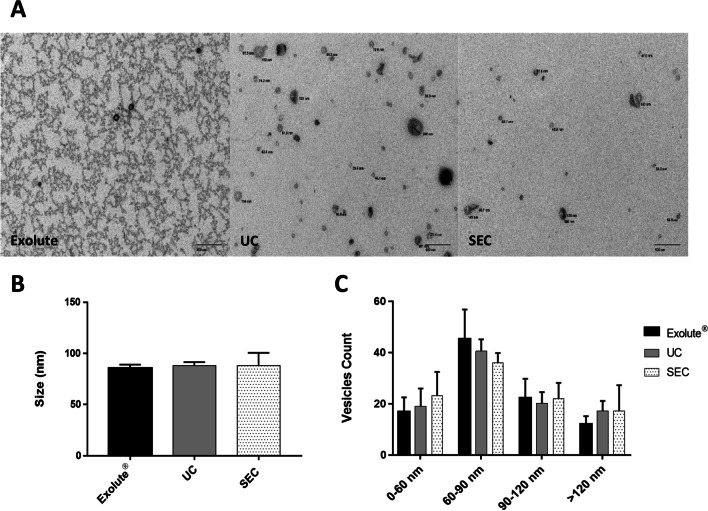
EVs characterization by TEM. **A** TEM images show EVs of the three methods used. **B** Mean size and **C** vesicle size distribution

To obtain an accurate determination of the size of the isolated EVs by the different isolation methods, and to confirm previous data obtained by NTA, a TEM analysis was performed. Membrane-limited vesicles were easily detected by TEM (Fig. 2A). Interestingly, whereas UC and SEC-isolated EVs preparations showed a clear background in most preparations, the presence of a “dense background smudge” was noted in Exolute® samples, consistent with the presence of protein aggregates, as it has been previously described by Karimi et al. [32]. TEM analysis showed that EVs displayed similar sizes between the three isolation methods, with a median size around 90 nm [ranged between 80.2 and 86.3; 84–91.5 and 78.7–105.7 for Exolute®, UC and SEC, respectively (*p*-value = 0.65)] (Fig. 2B). Differences in EVs size distribution were detected, where vesicles in the range of 60 to 90 nm represented the main isolated population (around 40% of total). Nevertheless, SEC-isolated EVs had a broader size distribution than those isolated with Exolute® and UC. In fact, Exolute® seemed to favor the isolation of medium size EVs (60–120 nm) in comparison with SEC and UC (Fig. 2C).

### Comparative marker analyses by AlphaScreen™ Technology

**Fig. 3:**
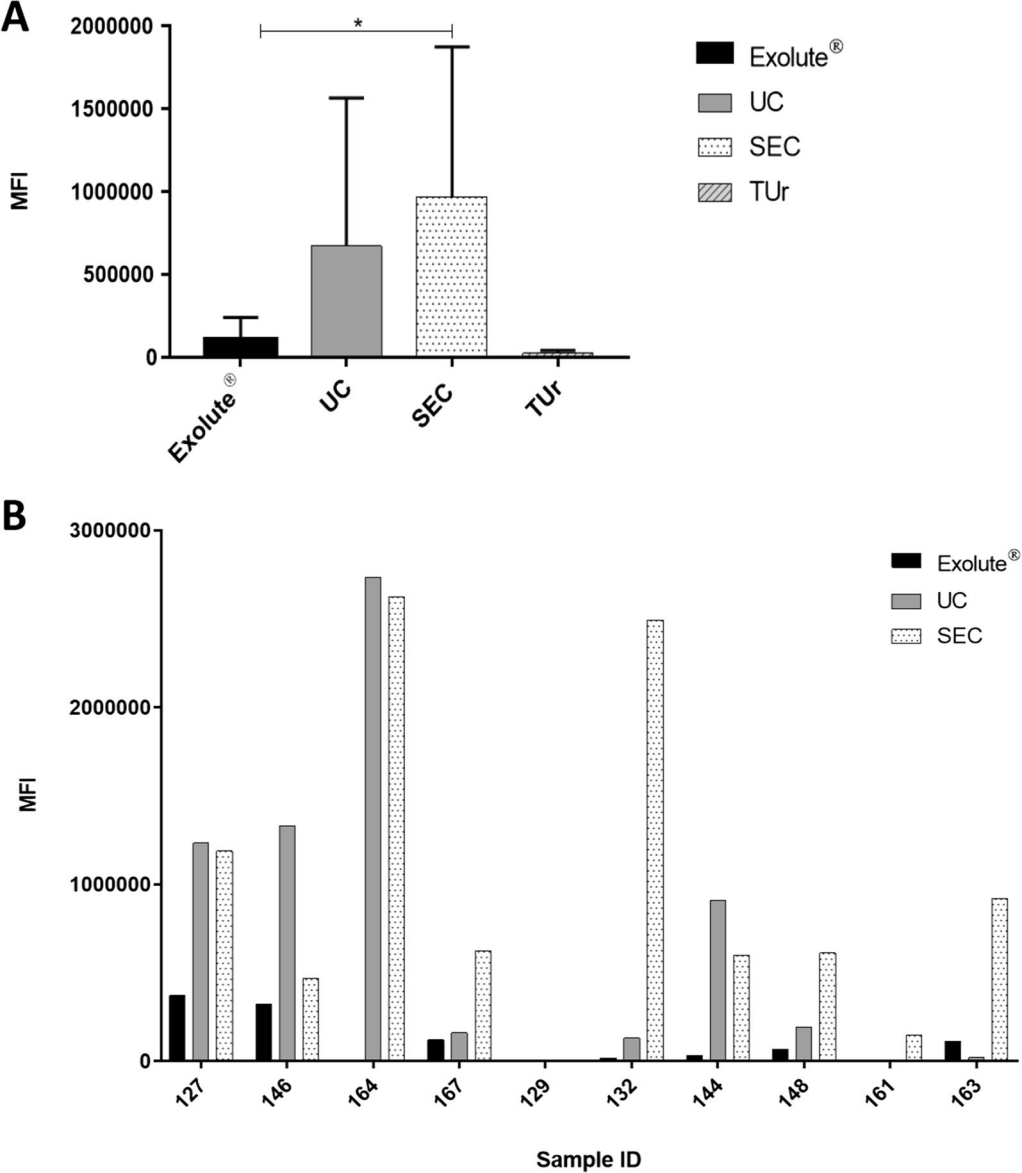
AlphaScreen™ Technology analysis. **A** CD9 and CD 63 tretaspanins have been used to label EVs. A higher performance has been obtained by the three isolation methods of EVs compared to total urine (TUr), being SEC the method with the highest performance followed by UC and Exolute®. (*p-value < 0.05). **B** Luminiscent levels shows variability in CD9 and CD63 EVs signal in each sample

Human EVs commonly present tetraspanin proteins at their surface, such as CD63, CD81 and CD9 [22, 33], which have been also detected in EVs from urine [32]. An adaptation of the amplified luminescent proximity homogeneous assay (ALPHA) technology [34] was used to detect two of these tetraspanins, CD9 and CD63, in EVs smaller than 200 nm. Our data showed that CD9 and CD63 were highly enriched in EVs from almost all the samples (with the exception of sample ID 129, which provided very low signals) when compared to total urine (TUr), confirming that the three methods were useful to isolate CD9 and CD63 positive EVs. As shown in Fig. 3A, SEC is the most efficient method in providing a reliable concentration of CD9 and CD63 positive EVs (9.7 × 10^5^ ± 9.1 × 10^5^), followed by UC (6.7 × 10^5^ ± 8.9 × 10^5^) and Exolute® (1.1 × 10^5^ ± 1.3 × 10^5^), respectively (*p*-value = 0.0233). Interestingly, when luminescent data are disaggregated per sample and isolation method (Fig. 3B) it can be observed that SEC provides sufficient signal in almost every sample, whereas there is a broad variability in CD9 and CD63 combined signals in UC-EVs. Thus, whereas UC-EVs had the higher mean signal in four samples, EVs isolated from the rest provided low signals (below 200,000), SEC-EVs showed the higher mean signal in 6 out of 10 patients, but only 2 patients provided signals below 200,000. On the other hand, no differences were detected between PCa and HDs (Additional file 1: Figure S2).

### Evaluation of the isolation method by HTG EdgeSeq miRNA whole transcriptome assay

**Fig. 4:**
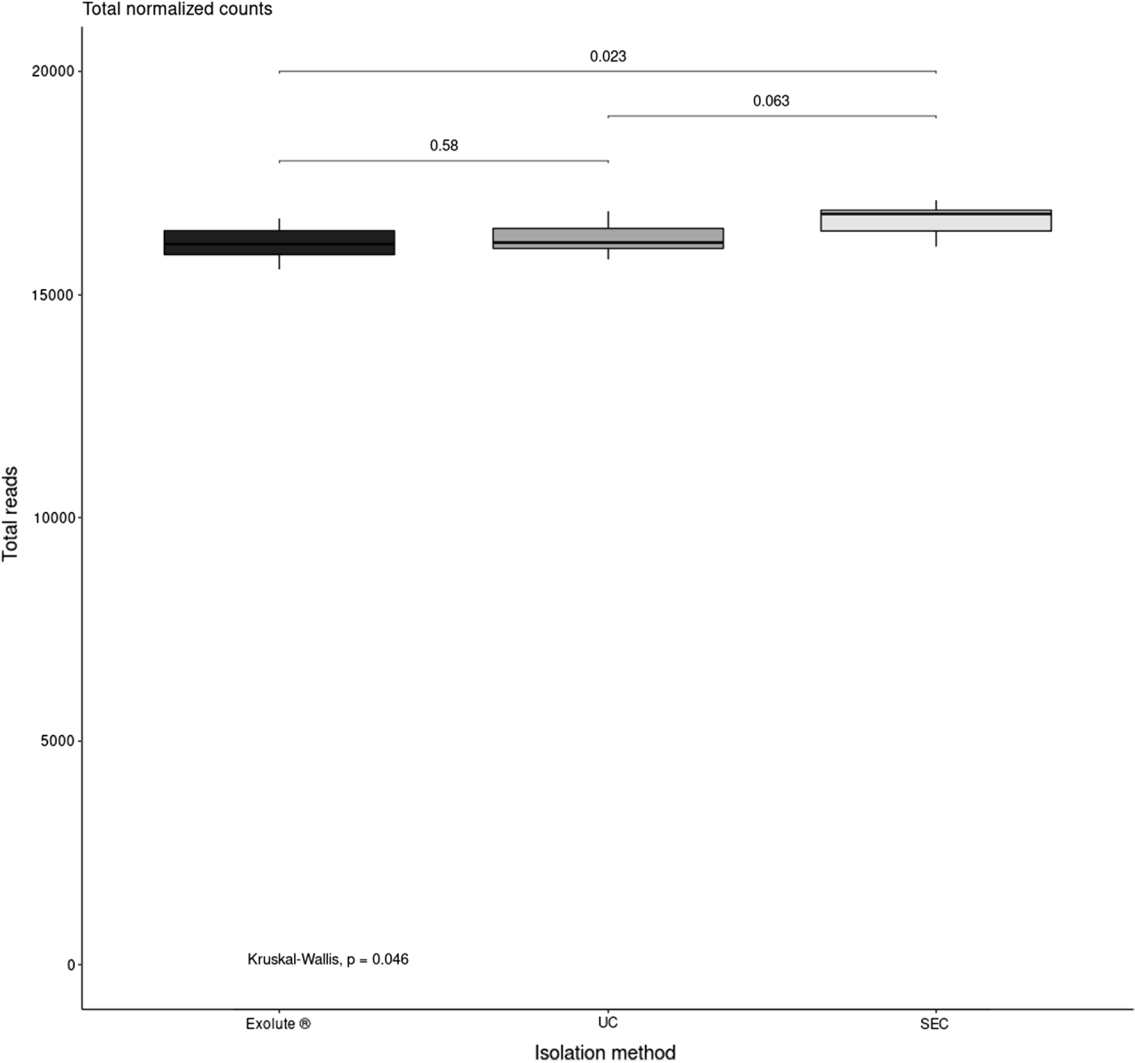
Box plot of the sum of the normalized miRNA counts per EVs isolation methods. SEC reported a higher count reads in comparison to UC (*p*-value = 0.063) and Exolute® (*p*-value = 0.023)

A whole miRNA transcriptomic assay was performed on the isolated EVs using the WTA panel on an HTG EdgeSeq Sytem (HTG Molecular Diagnostics). miRNA expression levels were quantified by NGS. Median of the sum of the normalized miRNA counts was higher in EVs isolated from SEC [16814 (range: 15,650–17,121)], followed by UC [16260 (range: 15,796–16874], and Exolute® [16137 (range: 15,575–16,710)] (*p*-value = 0.046) (Fig. 4).

Heatmap and PCA plots show that cases were not aggregated based on their miRNA expression profiles as would be expected, but they were classified more dependent of the EVs isolation method (Additional file 1: Figure S3). Despite this, correlations of miRNA expression levels in each case between the three EVs isolation methods were statistically significant (Table 2, Additional file 1: Figures S4–S13). However, in global, the correlation was better between UC and SEC [Median R^2^ = 0.8 (range: 0.62–0.91)] than any of these methods with Exolute® (Table 2).

**Table 2:** Coefficient correlation values (R^2^) between the three EVs-isolation procedures

Sample ID	Type of sample	UC-SEC	SEC-Exolute®	UC-Exolute®
127	PCa patient	0.77	0.66	0.73
129	PCa patient	0.65	0.64	0.70
132	PCa patient	0.82	0.57	0.67
144	PCa patient	0.84	0.77	0.75
146	PCa patient	0.91	0.76	0.75
148	PCa patient	0.72	0.79	0.71
161	HDs	0.62	0.67	0.77
163	HDs	0.78	0.81	0.69
164	HDs	0.83	0.63	0.64
167	HDs	0.82	0.63	0.69
Median (range)		0.8 (0.62–0.91)	0.67 (0.57–0.81)	0.71 (0.64–0.77)
Average (SD)		0.78 (0.09)	0.69 (0.08)	0.71 (0.04)

SD, standard deviation; HDs, healthy donors; PCa, prostate cancer

The differential expression analysis (DEA) between PCa cases and HDs for each EVs isolation method showed that a total of 21 and 3 miRNAs were differentially expressed (*p*-adjusted < 0.1) between groups for SEC and UC, respectively. However, no miRNAs were discriminated in the case of Exolute®. Remarkably, only miR-8052 matched between the SEC and UC miRNA sets (Additional file 1: Figure S14).

**Fig. 5:**
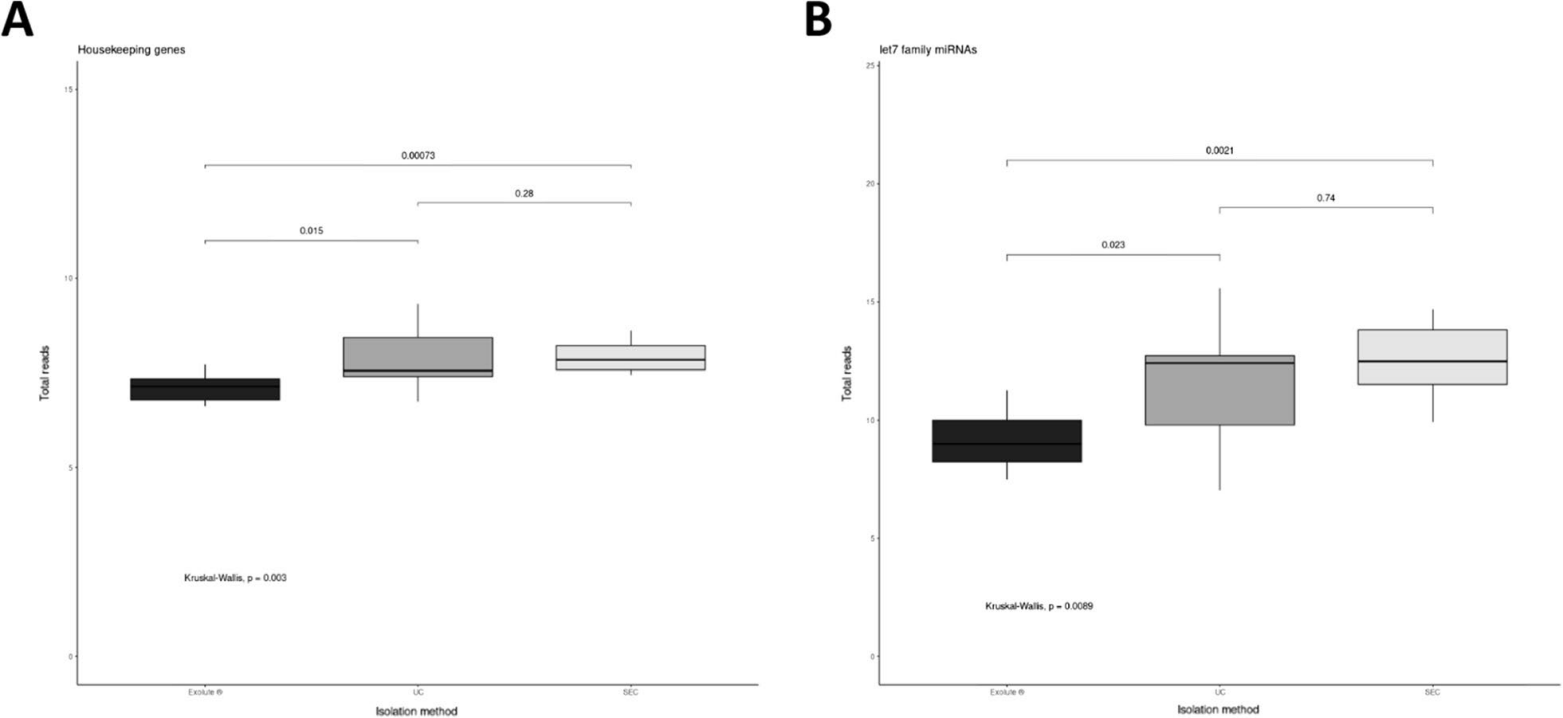
Box plots showing the median expression values for different EVs isolation methods of: **A** Twelve housekeeping genes included in the WTA panel (probes for the following genes are included: *B2M, GAPDH, PPIA, RNU47, RNU75, RNY3, RPL19, RPL27, RPS12, RPS20, SNORA66 YWHAZ*); **B** Let-7 family of miRNAs (n = 15). In both cases, clear differences between UC and SEC with Exolute® were appreciated

Interestingly, the evaluation of two sets of RNAs (housekeeping genes and *Let-7* miRNA family) from different EVs isolation methods showed significant differences between UC and SEC with Exolute® (Fig. 5), suggesting that Exolute® method offers the lowest performance from the biological point of view.

Lastly, the overall miRNA expression was divided into different quartiles (q1 < q2 < q3 < q4), and a correlation with the EVs isolation method used was performed for each quartile, indicating that those miRNAs with lower expression levels would be compromised depending on the type of isolation method used, as can be subtracted from the R^2^ values of the different quartiles (Additional file 1: Figure S15).

## Discussion

EVs have been postulated as a valuable source of potential biomarkers in PCa that at some point would complement or replace the routine diagnostic procedures [15, 35]. Urinary EVs take special relevance since their cargo reflect changes in the cellular biology of the tumour during progression and can be isolated by non-invasive procedures. However, translation of these biomarkers into the clinical setting is not exempt of limitations, including the irreproducibility of results as one of the most important [36]. In this regard, EVs isolation and characterization approaches still constitute a scientific challenge [23]. Hence, with the aim of deepening in the knowledge of EVs isolation methods, we evaluated three different methodologies including the classical UC and SEC, as well as a commercial eVs isolation kit (Exolute®). EVs characterization was performed using NTA, TEM, spectrophotometry (Nanodrop), AlphaScreen™ Technology and whole miRNA transcriptome expression analysis with the EdgeSeq System (HTG Molecular Diagnostics). For this purpose, a series of urines collected from 10 individuals (6 PCa patients and 4 HDs) were analysed with each isolation method.

Absorbance measurements showed similar protein concentrations in UC and SEC; however, a significant increase was noticed in Exolute® samples (Additional file 1: Figure S1A). This effect can be explained by TEM analysis, in which preparations from EVs obtained with Exolute® showed a background of precipitated proteins (Fig. 2A) consistent with the presence of protein aggregates, as it has been previously described [37]. NTA evaluation showed that UC provided the highest number of particles per mL and particles per µg protein ratio, in comparison with SEC and Exolute®, suggesting a higher EV yield obtained by this technique (Fig. 1A, C). Besides that, a significant increase in size of particles obtained by SEC was noticed (~ 190 nm), when compared to the other EVs isolation procedures (~ 165 nm) (Fig. 1B). However, no differences in size were appreciated when EVs were analyzed by TEM with a median in size of around 90 nm (range: 30–200 nm). Interestingly, approximately 95% of EVs were ranged from 30 to 120 nm, and around 40% were between 60 and 90 nm of diameter (Fig. 2C). These findings correlate with previous reports showing that the size of urine EVs varies from 30 to 100 nm [38–41]. Discordances between NTA and TEM herein reported may be due to different aspects including: the difficulty of NTA to resolve EVs aggregates (a correct dilution of the sample is crucial to avoid this) [42]; the limitation of NTA in detecting particles which dimeter is lower than 100 nm [43] and finally, the size overestimation of NTA [44, 45]. Additionally, and as mentioned above, TEM analyses revealed high variability in EVs yield obtained by Exolute®, with some samples showing the presence of protein aggregates that would explain the highest protein content of the spectrophotometric analysis (Fig. 2A). Interestingly, SEC-isolated EVs had a broader size distribution than those isolated with Exolute® and UC, which could be due to the growing evidence that SEC minimally alters the physical properties of EVs, whereas UC might cause vesicle rupture or fusion with proteins because of the high speed used in centrifugations [46]. No significant differences in particle concentration and size (measured by both NTA or TEM) were detected between PCa samples and HDs (Fig. 1D) which is in accordance with previous studies [38].

Once characterized through NTA and TEM, EVs were analysed with AlphaScreen™ Technology, a strategy lately used to improve the typical immunoassays [34] through the simultaneous detection for two specific EV-tetraspanins, CD9 and CD63 [22, 33] and designed to specifically detect EVs lower than 200 nm. Our AlphaScreen data revealed that all three isolation methods obtained CD9 and CD63 positive EVs, as can be appreciated in Fig. 3A where luminescent signal was higher in purified EVs compared to crude urine. Furthermore, SEC provided the highest luminescent intensity followed by UC and Exolute®, which luminescent intensity was significantly lower. Interestingly, a high signal variation was noted among the analysed cases, especially in Exolute® and UC isolated EVs, for which in some cases the luminescent signal was low or null, whereas SEC isolated EVs provided a measurable signal in most of the cases (Fig. 3B). These differences would be related to those herein noticed with regards EVs size distribution, or as suggested by some reports, as consequence of differences in the membrane proteomic content of small and large EVs [47]. Moreover, EVs rupture due to UC high-speed centrifugations [46] or the reported variability on EVs yield depending on the equipment and operator technical variability could have affected the results [48]. Hence, and according to the AlphaScreen™ Technology, our results highlight SEC as the most efficient method to isolate CD9 and CD63 positive microvesicles, followed by UC and Exolute®, respectively. Like the other characterization methods, no differences of luminescent signal were appreciated between EVs isolated from PCa patients and HDs for any of the isolation methods tested.

Forward characterization of EVs was carried through a whole miRNA transcriptomic analysis by using one of the newest and most reproducible RNA quantification platforms, the EdgeSeq Technology (HTG Molecular Diagnostics), currently used in many studies [49, 50]. Among the advantages of this system are that it does not require an RNA-extraction step which, reduces the extraction-associated data bias and sample loss; and the low input of sample necessary for being analysed [51]. Our results have shown that the sum of the normalized miRNA counts was higher in EVs isolated from SEC, followed by UC and Exolute® (Fig. 4), suggesting that the isolation methods influence on the yield of the transcriptomic analysis. Many -omic studies have been found to be highly dependent on the EVs isolation procedures, so that different methods produce EVs and EV sub-fractions of variable homogeneity, which makes difficult to extrapolate findings between different studies of EVs [7]. This is in line with the results we have obtained, in which samples were classified based more on the EVs isolation method than on their origin (PCa patients or HDs) (Additional file 1: Figure S3). Despite this, correlation of miRNA profiles between the different isolation approaches was high in all cases, being better between UC and SEC (Table 2). Interestingly, when miRNA expression was divided into quartiles, the best correlation coefficients (R^2^) were obtained in q4 (higher number of miRNAs) with a R^2^ = 0.97 between SEC and UC, the correlation in q2 and q1 being lower (Additional file 1: Figure S15). To demonstrate that the isolation procedures influence at biological level, two sets of RNA were evaluated: the housekeeping genes provided by the assay and the *Let-7* family of miRNAs. This approach showed significant differences with regards number of reads for any of the two RNA sets of UC and SEC with Exolute® (Fig. 5), indicating that Exolute® provides the lowest performance from the biological point of view.

DEA between PCa cases and HDs for each EVs isolation method showed that a total of 21 and 3 miRNAs were differentially expressed between groups for SEC and UC respectively, and none for Exolute®. The only miRNA that was differentially expressed in both methods was miR-8052 [52], a miRNA not previously described in PCa but in serum from sepsis patients with different outcomes [53]. Remarkably, two of the 3 miRNAs differentially expressed in EVs isolated by UC from urine of PCa patients were miR-142-5p and miR-223-3p, two miRNA that have been recently described in EVs from urine isolated also by UC as non-invasive PCa diagnostic biomarkers [54].

Although our study sample is limited, and further studies need to be addressed, our results point out to what other authors have suggested, the need of methodological standardization of EVs isolation and characterization to guaranty the success and reproducibility of the subsequent analysis, especially for clinical settings, where a large number of samples should be analysed [55–57]. In this line, we suggest developing a codification system focused on the EV isolation and characterization variables like the SPREC codification system for pre-analytical conditions [25] that we have introduce with our samples (Table 1) and that provides information on the handling of biological specimens before analysis, another critical point that not always comprehensively considered [58, 59].

## Conclusion

Definitively, our study highlights the impact that the EV isolation method may have on the analytical results, as differences in the yield, purity, and status of the obtained EVs might have a great influence in the clinical setting. For this reason, methodological standardization in the isolation and subsequent analysis of EV is crucial to guaranty the reproducibility necessary for their implementation in different clinical scenarios.

## Supplementary Information

**Additional file 1**. Supplementary material.

## Data Availability

Data and materials available upon request.

## Abbreviations

CREC: Clinical Research Ethics Committee
DEA: Differential expression analysis
DRE: Digital rectal examination
EVs: Extracellular vesicles
FIVO: Fundación Instituto Valenciano de Oncología
HDs: Healthy donors
miRNA: MicroRNA
NTA: Nanoparticle tracking analysis
PBS: Phosphate-Buffered Saline
PCa: Prostate cancer
PCR: Polymerase chain reaction
PSA: Prostate-specific antigen
qPCR: Quantitative PCR
RNA: Ribonucleic acid
RT: Room temperature
SCSIE: Central Service for Experimental Research
SEC: Size exclusion chromatography
SPREC: Sample PREanalytical Code
TEM: Transmission electron microscopy
UC: Ultracentrifugation
WTA: Whole Transcriptome Assay
