# Supplementary material for: Isolation and characterization of urine microvesicles from prostate cancer patients: different approaches, different visions
Source: BMC Urol. 2021 Sep 27;21:137. doi: 10.1186/s12894-021-00902-8 (PMC8477576; doi:10.1186/s12894-021-00902-8)
Supplement: Supplementary file 1 — Additional file 1. Supplementary material. [file 12894_2021_902_MOESM1_ESM.docx]

Supplementary material

**Isolation and Characterization of Urine Microvesicles from Prostate Cancer Patients: Different Approaches, Different Visions**

María García-Flores^1,2#^, Christian M. Sánchez-López^3,4#^, Marta Ramírez-Calvo^1^, Antonio Fernández-Serra^1^, Antonio Marcilla^3,4,¶^, José Antonio López-Guerrero^1,2,5, ¶*^

^1^ Laboratory of Molecular Biology, Fundación Instituto Valenciano de Oncología, Valencia 46009, Spain. MGF: [mgarciaf@fivo.org](mailto:mgarciaf@fivo.org), ORCID: 0000-0002-2232-5717. MRC: [mramirezc@fivo.org](mailto:mramirezc@fivo.org), ORCID: [0000-0002-7179-5360](mailto:%200000-0002-7179-5360). AFS: [afernandez@fivo.org](mailto:afernandez@fivo.org), ORCID: [0000-0003-0851-5239](mailto:0000-0003-0851-5239). JALG: [jalopez@fivo.org](mailto:jalopez@fivo.org), ORCID: [0000-0002-7369-8388](https://orcid.org/0000-0002-7369-8388).

^2^ IVO-CIPF Joint Research Unit of Cancer, Príncipe Felipe Research Center (CIPF), Valencia 46012, Spain.

^3^ Àrea de Parasitologia, Departament de Farmàcia i Tecnologia Farmacèutica i Parasitologia, Universitat de València, Burjassot, Valencia 46000, Spain. CSL: [chsanlo@alumni.uv.es](mailto:chsanlo@alumni.uv.es), ORCID: 0000-0001-6062-5940. AM: [Antonio.Marcilla@uv.es](mailto:Antonio.Marcilla@uv.es), ORCID: 0000-0003-0004-0531.

^4^ Joint Research Unit on Endocrinology, Nutrition and Clinical Dietetics, Health Research Institute La Fe, Universitat de Valencia, Valencia 46100, Spain.

^5^ Department of Pathology, School of Medicine, Catholic University of Valencia “San Vicente Mártir”, Valencia 46001, Spain.

# Equal contribution.

^¶^ Co-senior authors.

***** Corresponding author.

E-mail: [jalopez@fivo.org](mailto:jalopez@fivo.org) (JALG).

**
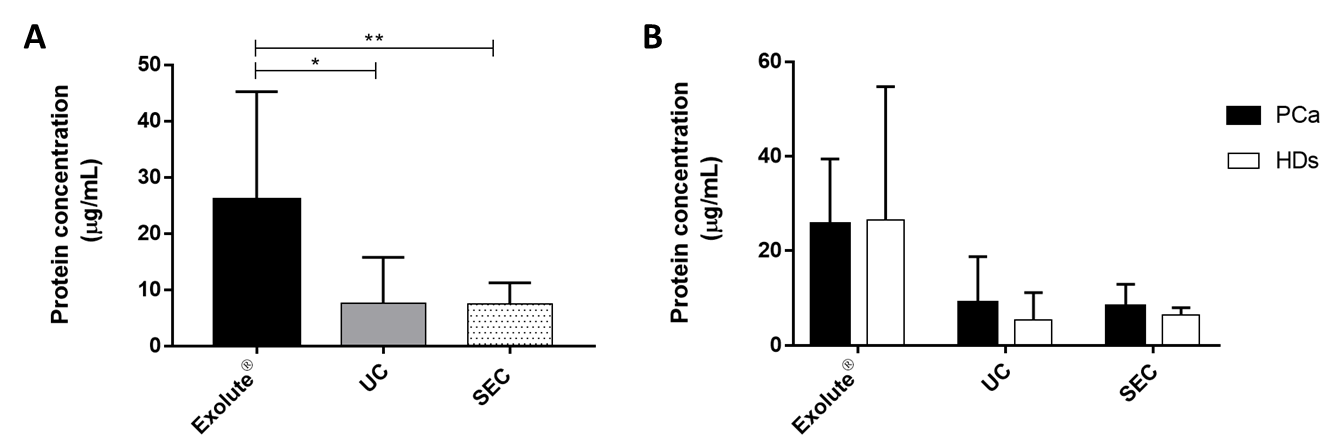
**

**Figure S1 Distribution of protein quantification.** (**A**) Spectrophotometric quantification at 280 nm showing similar protein concentrations between UC and SEC. Exolute^®^ showed the highest protein concentration. (**B**) No differences in protein concentration were observed between PCa and HDs in any of the three isolation methods. (*, *p*-value <0.05; **, *p*-value <0.001).

**
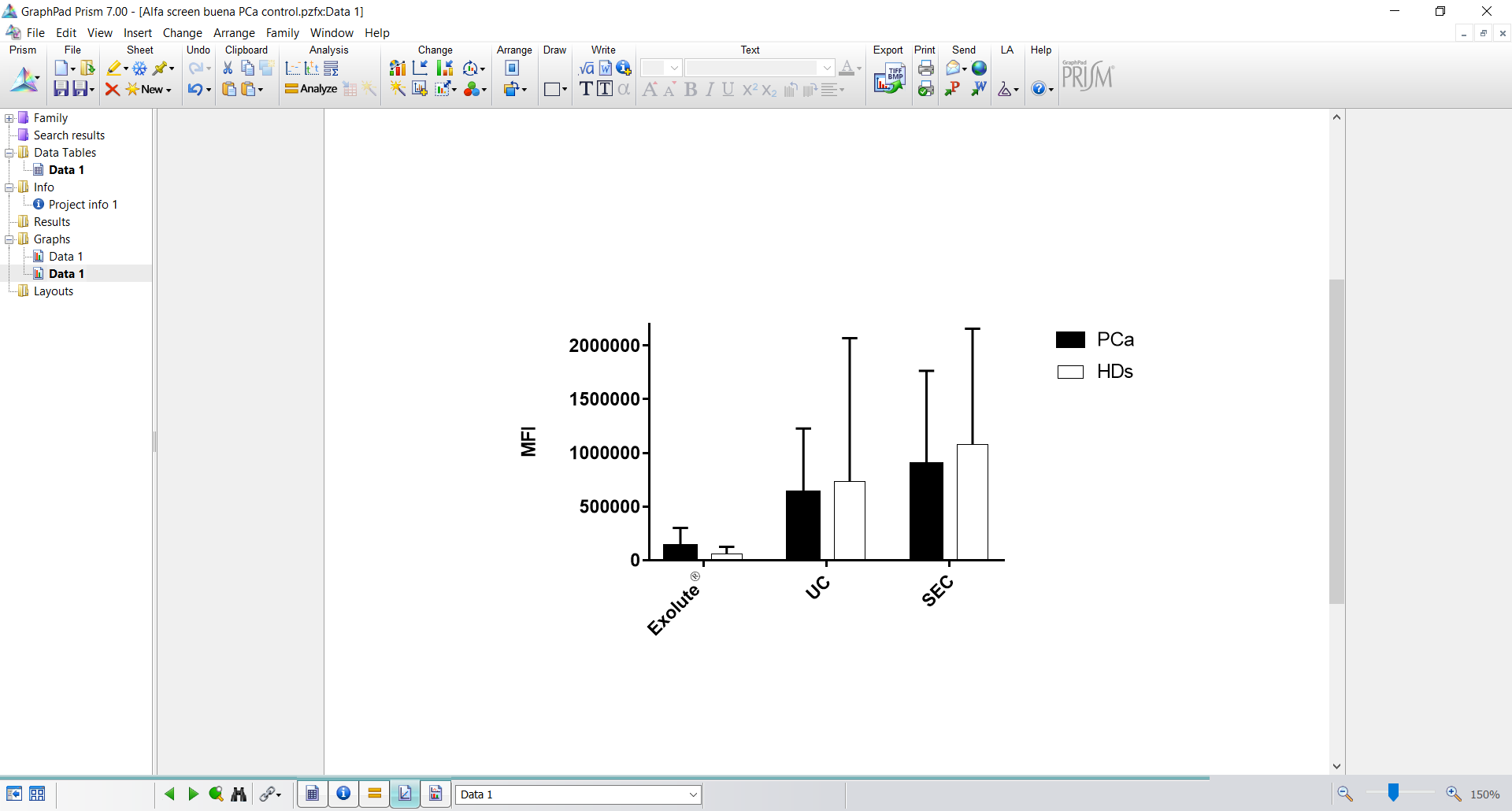
**

**Figure S2 AlphaScreen™ Technology analysis between groups.** Luminiscent levels shows that there are no significant differences between CaP and HDs.


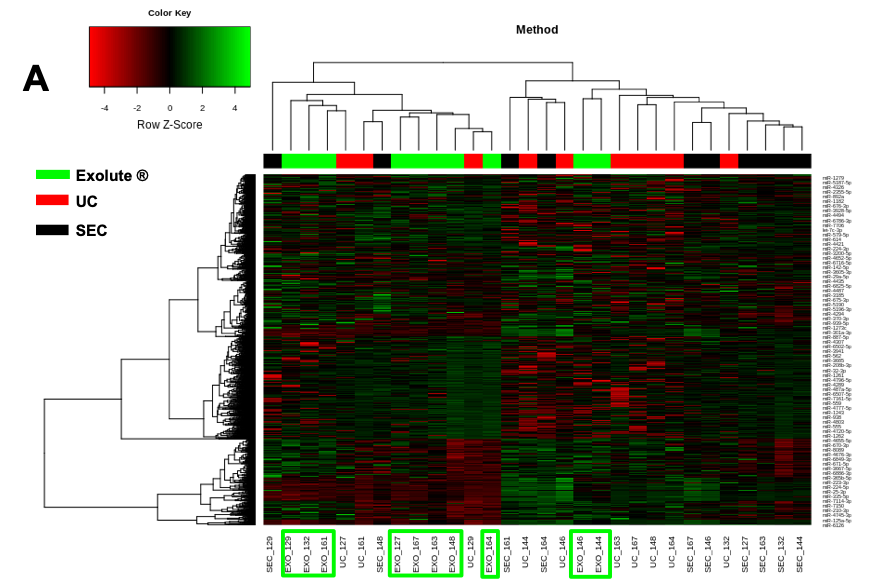

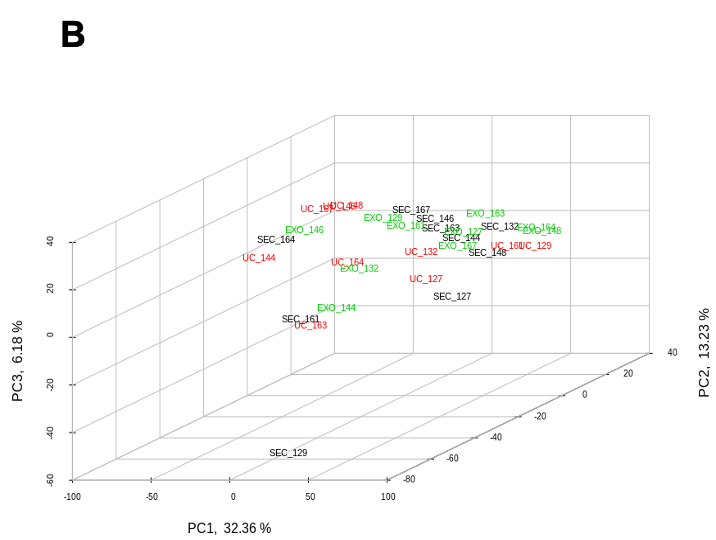


**Figure S3** Heatmap (**A**) and PCA (**B**) plots showing the distribution of cases depending on their miRNA expression profile. As can be appreciated, classification of cases is mainly dependent on the type of EVs isolation methods and not because of the similarities of the miRNAs content of the cases.


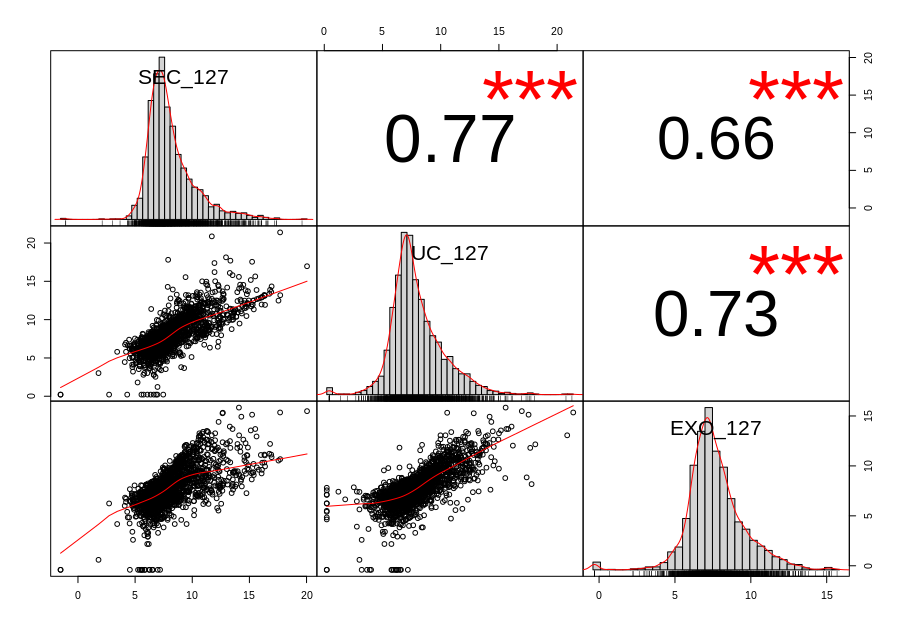


**Figure S4** Pearson correlation (R2) of the miRNA expression levels between the three EVs-isolation methods for sample ID127. (***, *p*-value <0.0001).


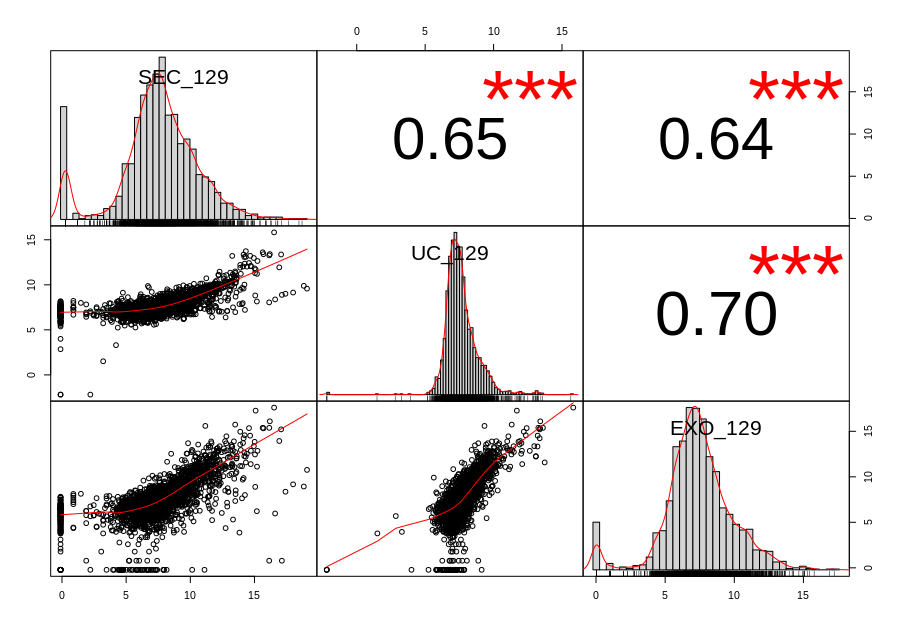


**Figure S5** Pearson correlation (R^2^) of the miRNA expression levels between the three EVs-isolation methods for sample ID129. (***, *p*-value <0.0001).


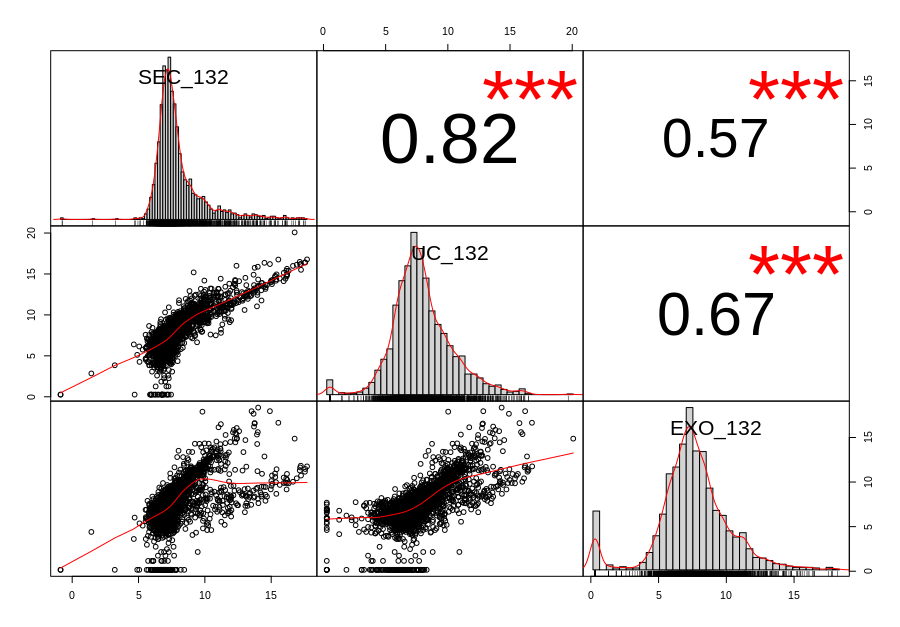


**Figure S6** Pearson correlation (R^2^) of the miRNA expression levels between the three EVs-isolation methods for sample ID132. (***, *p-*value <0.0001).


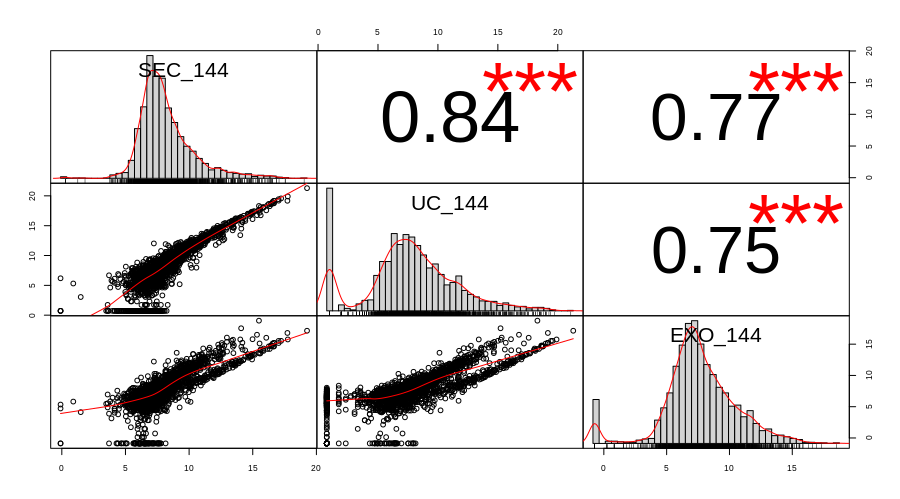


**Figure S7** Pearson correlation (R^2^) of the miRNA expression levels between the three EVs-isolation methods for sample ID144. (***, *p*-value <0.0001).


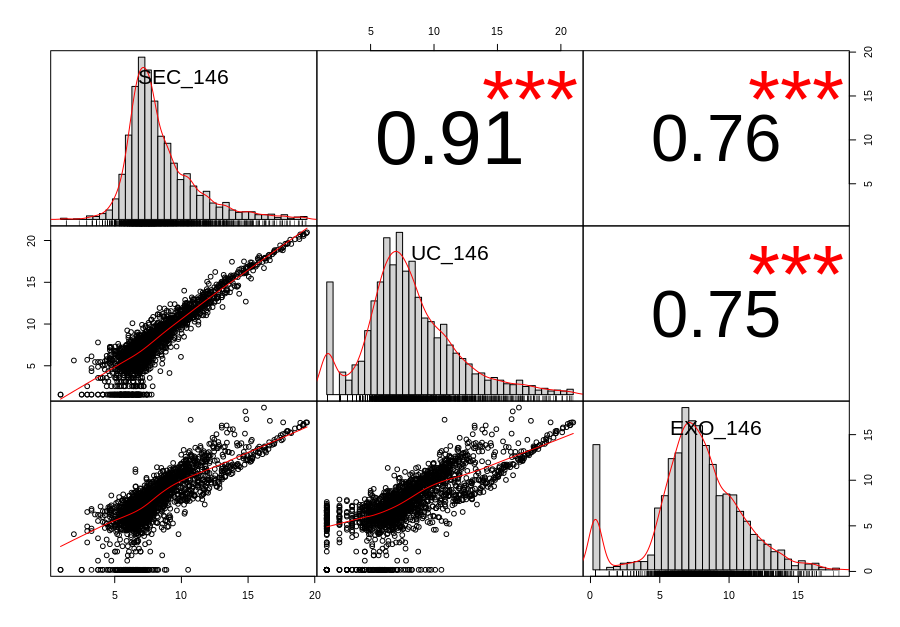


**Figure S8** Pearson correlation (R2) of the miRNA expression levels between the three EVs-isolation methods for sample ID146. (***, *p*-value <0.0001).


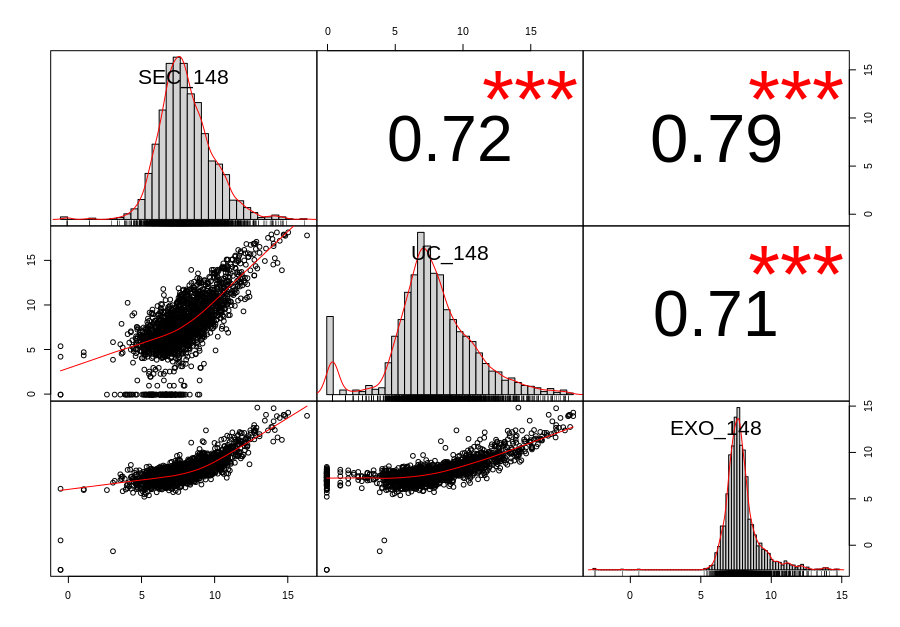


**Figure S9** Pearson correlation (R2) of the miRNA expression levels between the three EVs-isolation methods for sample ID148. (***, *p*-value <0.0001).


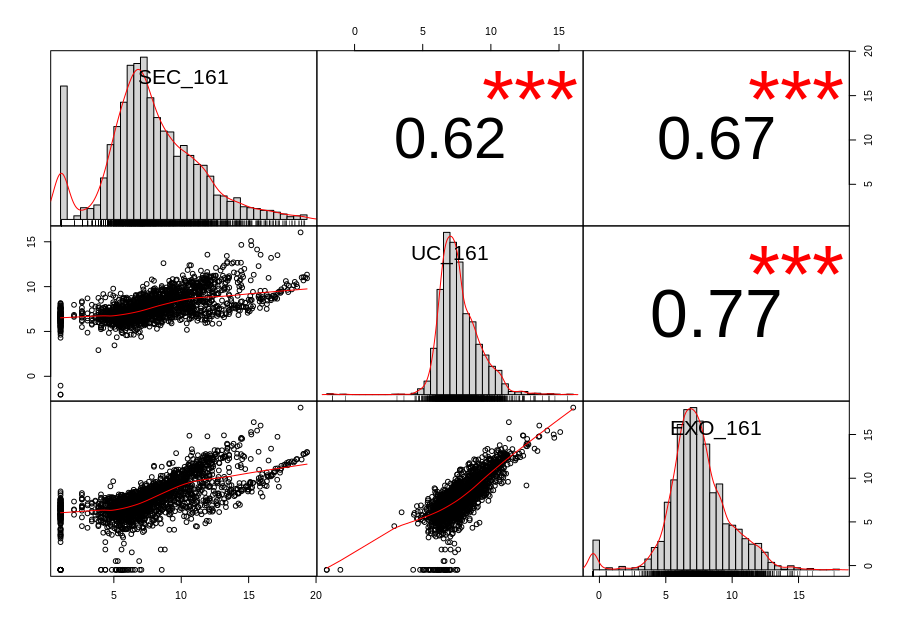


**Figure S10** Pearson correlation (R2) of the miRNA expression levels between the three EVs-isolation methods for sample ID161. (***, *p*-value <0.0001).


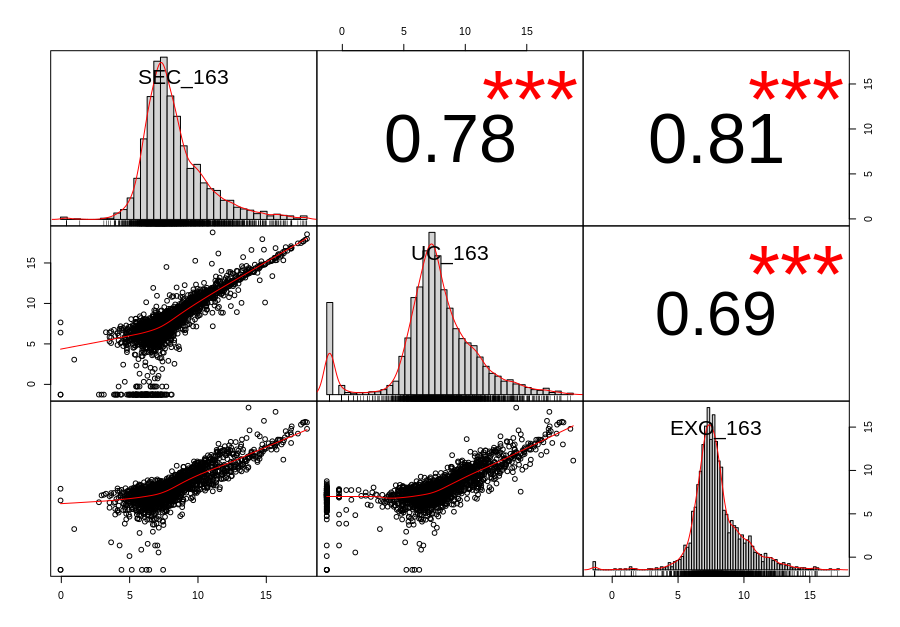


**Figure S11** Pearson correlation (R2) of the miRNA expression levels between the three EVs-isolation methods for sample ID163. (***, *p*-value <0.0001).


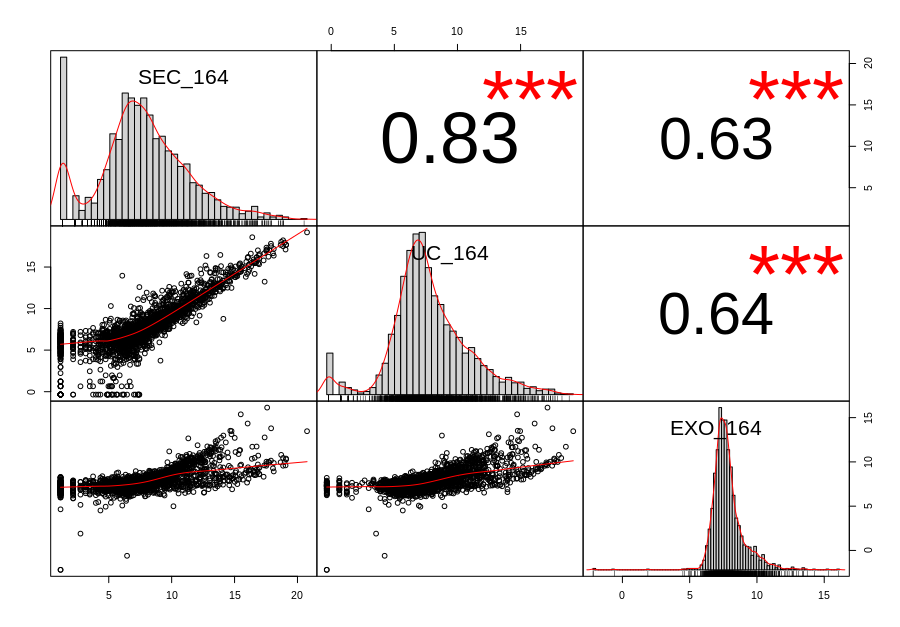


**Figure S12** Pearson correlation (R2) of the miRNA expression levels between the three EVs-isolation methods for sample ID164. (***, p-value <0.0001).


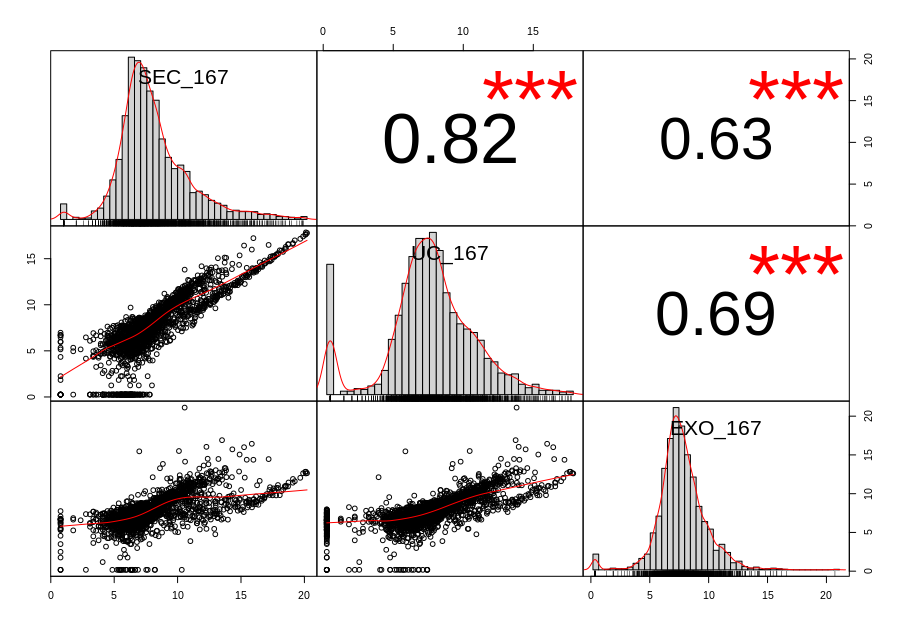


**Figure S13** Pearson correlation (R2) of the miRNA expression levels between the three EVs-isolation methods for sample ID167. (***, *p*-value <0.0001).

**Figure S14** Differential expression analysis (DEA) of urine EVs-miRNAs between PCa (n=6) and control (HDs) (n=4) cases. (**A**) Heatmap corresponding to the DEA on EVs isolated by SEC; (**B**) List of the 21 miRNAs differentially expressed between PCa and controls with an adjusted *p*-value < 0.1; (**C**) Heatmap corresponding to the DEA on EVs isolated by UC; (**D**) List of the 3 miRNAs differentially expressed between PCa and controls (adjusted *p*-value < 0.1); (E) Venn diagram showing that only miR-8052 matches between the miRNA significant DEA sets between SEC and UC.

**Figure S15** Pearson correlation (R2) of the miRNA expression levels between the three EVs-isolation methods divided into quartiles (q1<q2<q3<q4). (**, *p*-value <0.001; ***, *p*-value <0.0001).
